# Physiotherapists’ Clinical Reasoning and Therapeutic Strategies in Fibromyalgia: A Qualitative Interpretative Study

**DOI:** 10.3390/jcm15156099

**Published:** 2026-08-05

**Authors:** Daniel Sanjuán-Sánchez, Paula Cordova-Alegre, José Lesmes Poveda-López, Oriol Martínez-Navarro, Francesc Rubí-Carnacea, Erica Briones-Vozmediano

**Affiliations:** 1 Facultad de Ciencias de la Salud, Universidad San Jorge, Villanueva de Gállego, 50830 Zaragoza, Spain; dsanjuan@usj.es (D.S.-S.); jlpoveda@usj.es (J.L.P.-L.); 2 Departament d’Infermeria i Fisioteràpia, Universitat de Lleida, 25198 Lleida, Spain; oriol.martinez@udl.cat (O.M.-N.); francesc.rubi@udl.cat (F.R.-C.); erica.briones@udl.cat (E.B.-V.); 3 Grup de Recerca Societat, Salut, Educació i Cultura (GESEC), Universitat de Lleida, 25198 Lleida, Spain; 4 Grup de Recerca de Cures en Salut (GRECS), IRBLleida (Institut de Recerca Biomèdica de Lleida, Fundació Dr. Pifarré), 25198 Lleida, Spain

**Keywords:** fibromyalgia, physiotherapy, pain neuroscience education, therapeutic alliance, exercise therapy, self-management, qualitative research, biopsychosocial model

## Abstract

**Background/Objectives:** Fibromyalgia (FM) is a chronic pain condition characterized by widespread pain, fatigue, sleep disturbances, and psychosocial challenges. Although physiotherapists play a key role in FM management, little is known about how biopsychosocial principles are applied in clinical practice. This study explored physiotherapists’ clinical reasoning, therapeutic strategies, and approaches to promoting adherence and functional recovery in FM. **Methods:** A qualitative study was conducted within a constructivist–interpretative paradigm. Thirteen physiotherapists with experience in FM management across different healthcare settings in Spain participated in semi-structured interviews. Participants were recruited through purposive and snowball sampling. Interviews were audio-recorded, transcribed verbatim, and analyzed using reflexive thematic analysis following Braun and Clarke’s framework. Methodological rigor was ensured through reflexive engagement among researchers with differing backgrounds, reflexive practice, and adherence to COREQ guidelines. **Results:** Three overarching themes and seven sub-themes were identified. Clinical validation emerged as a key strategy to reduce uncertainty and facilitate engagement. Therapeutic goals were progressively shifted from pain reduction toward function, autonomy, and participation. Pain neuroscience education and individualized exercise were identified as the main therapeutic strategies, while group-based interventions promoted social support, motivation, and adherence. Lifestyle recommendations, including stable routines, social participation, and work adaptation, complemented treatment. **Conclusions:** Physiotherapists perceive FM management as a multidimensional process extending beyond symptom reduction. Validation, pain neuroscience education, individualized exercise, and group-based support appear to facilitate adherence, self-management, and functional recovery within a biopsychosocial framework.

## 1. Introduction

Fibromyalgia (FM) is defined as a chronic and multifactorial syndrome characterized by widespread musculoskeletal pain, persistent fatigue, sleep disturbances, cognitive impairment, and emotional distress [1,2,3,4].

FM has an estimated global prevalence between 2% and 3%, although some studies suggest ranges up to 6.6% [5,6]. This condition predominantly affects women and has a substantial impact on quality of life and healthcare costs [3,7]. Regarding the pathophysiology, FM is associated with central sensitization mechanisms and nociplastic pain, wherein the central nervous system amplifies pain signaling [8,9]. Furthermore, recent evidence points toward cytokine-mediated low-grade inflammation and potential stress-related neuroendocrine alterations [10,11].

This syndrome represents a critical biopsychosocial challenge that falls outside the traditional biomedical model [1]. As it cannot be verified through conventional radiological imaging or blood tests, FM is frequently labeled as a “contested” illness or an “observable illness without disease” [12,13]. This lack of objective biomarkers generates misunderstanding within the healthcare system, hindering patient legitimation, and profoundly affecting personal identity [1,14]. In this context, recovery cannot be understood under the biomedical lens of “tissue repair”, but rather as a process of reconstructing a dignified and meaningful life despite the limitations associated with the condition [1,15,16].

Physiotherapists play a central role in the management of FM. Non-pharmacological interventions such as tailored physical exercise (including strength, mobilization and aerobic exercise) and Pain Neuroscience Education (PNE) are recommended as first-line treatments [3,17,18,19,20]. Nevertheless, clinical practice in FM entails high emotional and clinical complexity; the professional must manage not only functional disability but also factors such as catastrophizing, kinesiophobia, and the risk of depression and anxiety [14,21,22]. This responsibility requires physiotherapists to transcend traditional technical training, often centered on tissue pathology. It also requires them to avoid a fragmented view of the patient, and to address the uncertainty surrounding the efficacy of current therapies [15,16,23].

To achieve meaningful rehabilitation, physiotherapists must transform into a “guiding partner”, establishing a genuine, authentic, and non-judgmental connection. This involves fostering a robust therapeutic alliance where decision-making power is shared, and the individual’s lived experience is validated [14,16]. The challenge lies in integrating scientific evidence with a patient-centered approach that helps individuals make sense of their pain and address life stressors, which may otherwise contribute to symptom persistence [16,24,25].

This complexity situates FM not only as a clinical condition but as a profound challenge for healthcare professionals, and particularly for physiotherapists, who must manage a clinical context marked by diagnostic uncertainty, subjective symptom presentation, and notable emotional demands [26,27,28]. Although many studies have explored patients’ experiences with FM, far less attention has been given to the perspectives of physiotherapists, despite their key role in long-term care [29,30]. Only a limited number of qualitative studies have explored physiotherapists’ experiences, often highlighting role uncertainty and perceived professional shortcomings [31].

Existing research has predominantly focused on the effectiveness of specific interventions, establishing a solid theoretical basis for the biopsychosocial management of FM [18,32]. However, a critical implementation gap persists. Despite growing consensus on what should be done, far less is understood about how these approaches are enacted in real-world clinical practice, where therapeutic relationships, contextual variability, and moment-to-moment clinical decisions shape the actual delivery of care [33,34]. This gap between theoretical frameworks and their clinical operationalization represents a crucial but underexplored area of inquiry, particularly given the complexity and unpredictability inherent in FM management.

Also, despite the growing body of literature regarding patients’ lived experiences with FM, there remains a significant gap in understanding how expert clinicians navigate the complexities of its long-term management. Specifically, little is known about the decision-making processes and psychosocial strategies employed by physiotherapists to foster treatment adherence in this population. For the purposes of this study, clinical validation is understood as acknowledging and legitimizing patients’ experiences, and therapeutic alliance as the collaborative relationship between the physiotherapist and the patient. Adherence is understood as sustained engagement with treatment and self-management strategies, and autonomy and participation as the patient’s ability to manage the condition and engage in meaningful daily activities [14]. This study addresses that gap by centering on physiotherapists, a professional group still markedly underrepresented in qualitative FM research, to elucidate how the biopsychosocial model is concretely operationalized within their everyday clinical reasoning and therapeutic strategies, rather than considered only at a theoretical level. Accordingly, the research question addressed was: How do physiotherapists perceive their clinical reasoning and accompanying strategies in FM care, and in what ways do clinical validation and PNE facilitate the transition from diagnostic uncertainty toward functional autonomy and patient adherence?

## 2. Materials and Methods

### 2.1. Study Design

A qualitative study was conducted within a constructivist–interpretative paradigm. This approach was chosen to explore the subjective meanings and clinical experiences of the participants. The study adhered to the COREQ (Consolidated Criteria for Reporting Qualitative Research) guidelines [35] to ensure methodological transparency and rigor (See Supplementary Materials).

Ethical approval was granted by the Hospital Arnau de Vilanova Research Ethics Committee (Ref: CEIC-2481). All participants were provided with comprehensive study information and signed a written informed consent form prior to their participation, which included permission for audio recording and the use of anonymized excerpts for publication. To protect anonymity, alphanumeric identifiers (e.g., PT1, PT2) were assigned to each participant.

### 2.2. Participants and Sampling

Participants were recruited using a purposive sampling strategy (*n* = 2), subsequently enriched by a snowballing technique (*n* = 11) to access clinicians with deep experiential knowledge. This dual approach facilitated the inclusion of key informants who provided a comprehensive overview of FM management across diverse regions of Spain, including the Northwest, Northeast, and Mediterranean areas. This geographical diversity ensured a broad perspective on different clinical and regional healthcare settings.

Initial participants (*n* = 2) were identified through professional networks and targeted searches based on their clinical expertise in chronic pain and FM. Expertise was not defined by years of general clinical practice, but by focused and continuous clinical exposure to FM management, meaning that participants with comparatively fewer years of overall practice could still be considered experienced if they had worked specifically and consistently with this population since early in their careers. These individuals then acted as gatekeepers, referring further eligible colleagues. This iterative process continued until data saturation was achieved. Specifically, meaning saturation was the criterion applied. Data collection continued until no new themes or conceptual meanings emerged from the physiotherapist’s narratives. Saturation was monitored iteratively throughout data collection by comparing the coding tree and thematic content across successive interviews. From interview 9 onward, increasing conceptual overlap was observed between new and previously identified codes, and although minor nuances continued to emerge in later interviews, no substantive new themes or interpretive dimensions were added to the existing thematic structure. Meaning saturation, reflecting stability in the core themes and their underlying meanings, rather than the complete absence of novel codes, was therefore considered achieved by interview 9 of 13, and was cross-checked independently by DS and EB and confirmed through team consensus during peer debriefing.

Inclusion criteria required participants to: (1) hold a degree in Physiotherapy obtained in Spain, (2) possess a minimum of two years of clinical experience, (3) have provided active treatment to patients with FM within the preceding 12 months, (4) be currently practicing within the Spanish healthcare system, including public, private, insurance-based, or patient association settings.

### 2.3. Data Collection and Context

Individual semi-structured interviews served as the primary data collection method, allowing for an in-depth exploration of participants’ clinical reasoning and lived professional experiences. An interview guide was developed based on a comprehensive literature review and aligned with the study’s objectives, utilizing the framework by Roitenberg and Shoshana [31] as a conceptual model. The guide comprised open-ended questions designed to elicit rich, narrative data regarding clinical management, perceived barriers and facilitators, and therapeutic decision-making in FM (Table 1). Interviews were conducted by the lead author (DS) between 2024 and 2025, with durations ranging from 45 to 75 min. All interviews were conducted in Spanish and analyzed in their original language. Illustrative quotes selected for inclusion in the Section 3 were subsequently translated into English by a professional translator unaffiliated with the research team and reviewed by the lead author (DS) to verify accuracy and ensure that clinical and conceptual nuances were preserved in translation.

Consistent with contemporary qualitative practice, data were collected through both face-to-face sessions and secure video calls (Microsoft Teams), depending on participant geographical location and preference. All interviews were conducted in private, quiet environments to foster a sense of psychological safety and encourage open disclosure. Each session was audio-recorded with explicit prior consent and subsequently transcribed verbatim for analysis. Data collection continued until thematic saturation was reached, with no new themes emerging from the interviews.

### 2.4. Data Analysis

Data analysis was conducted using reflexive thematic analysis following the framework by Braun and Clarke [36] (familiarization with the data, generation of initial codes, searching for themes, reviewing themes, defining and naming themes, and producing the report). Analysis was conducted at a latent level, moving beyond the semantic content of participants’ accounts to identify and interpret the underlying assumptions, meanings, and conceptual frameworks shaping their clinical experiences. This approach was consistent with the constructivist–interpretative paradigm guiding the study. The analytical process was primarily inductive. Initial codes were generated from close reading of the transcripts, reflecting broad areas of content (e.g., pain, therapist-patient relationship, active strategies) and were subsequently grouped into candidate themes through an iterative process in which each coder first organized codes into more specific sub-themes, before the research team jointly reviewed and reconciled the resulting thematic structure through discussion and abstraction. Themes were not understood as emerging naturally from the data, but as actively constructed through the researchers’ engagement with it, in line with Braun and Clarke’s reflexive approach. Reflexive notes were maintained throughout the analytical process (see Section 2.5). Qualitative data management, including both transcript data and reflexive notes, was facilitated by ATLAS.ti Web, version 24.

Initially, a preliminary coding tree was developed by EB and DS to organize raw data into initial meaning units, which were then iteratively refined into broader themes and sub-themes. Methodological rigor was supported by reflexive engagement among researchers with differing clinical and disciplinary backgrounds. Authors DS and EB independently coded and reviewed a subset of the transcripts and the analytical coding structure. Co-author PC contributed to thematic discussion and consensus meetings, while independent supervision was provided by FR; neither was directly involved in primary coding, contributing an additional interpretive perspective to the analytical process. Notably, while DS and PC approached the data as physiotherapists with prior clinical exposure to the phenomenon under study, EB brought a humanities perspective, enriching the interpretive depth of theme construction and broadening the range of disciplinary perspectives brought to bear on the data. Differences in interpretation were discussed during peer debriefing, which served to deepen and refine the analytical interpretation rather than to reach a single predetermined coding. To ensure the confirmability of the results, verbatim participant quotes were integrated into the findings, keeping the analysis grounded in the participants’ original experiences.

### 2.5. Reflexivity

The interviewer (DS) is a physiotherapist with 10 years of clinical experience including eight years working specifically with patients with chronic pain and has specific training in qualitative research methods. Co-author PC is also a physiotherapist with over 10 years of clinical experience and holds a PhD, having previously conducted doctoral research on migraine. Co-author EB has particular expertise in qualitative research methodology and in FM, having completed her own doctoral thesis on this condition from a social sciences perspective. This combination of clinical and methodological backgrounds shaped the research team’s engagement with the data, balancing clinical familiarity with FM management against a more critical, discipline-external reading of the material. Both DS and PC approached FM from a clinical standpoint that regards it as a legitimate, complex condition requiring biopsychosocial management, rather than a purely biomedical one, a perspective consistent with current pain science but which could also predispose the analysis toward findings that reinforce this framework. DS had a pre-existing professional relationship with some participants through shared professional networks in chronic pain and FM care; no hierarchical or supervisory relationship existed between DS and any participant. Confidentiality and voluntary participation were reinforced during data collection to minimize potential social desirability bias arising from this familiarity.

The research team’s clinical background informed an initial assumption that, while the biopsychosocial model is broadly endorsed by physiotherapists at a theoretical level, its translation into everyday clinical practice may not be consistent. This assumption was made explicit prior to analysis and revisited across the coding process through reflexive notes and peer debriefing to remain attentive to the ways it might orient interpretation toward implementation gaps. Reflexive notes, serving as an analytical decision log, were recorded after each interview and during coding to document emerging reactions, assumptions, and decisions shaping the analytic process; these notes were periodically discussed in team meetings to interrogate the interviewer’s clinical viewpoint and reduce its unchecked influence on theme construction.

## 3. Results

### 3.1. Participant Characteristics

A total of 13 physiotherapists participated in the study. The final sample represented a diverse range of clinical expertise and healthcare settings across Spain, including the public health system, private clinics, and patient associations. On average, participants possessed 11.31 years of professional experience in FM (range 1 to 29), ensuring a deep experiential understanding of FM management and providing a diverse range of clinical perspectives. The length of the mean interview was 53 min. Detailed demographic and professional characteristics for each participant are presented in Table 2.

### 3.2. Overview of Themes

The reflexive thematic analysis generated three overarching themes and seven sub-themes that illustrate the clinical journey and management strategies for FM from the professional perspective. These themes are closely interlinked, reflecting a transition from initial patient validation and cognitive re-orientation to the implementation of multimodal empowerment strategies. The thematic structure is summarized in Table 3, which presents a conceptual overview of the findings and synthesizes the core categories and their respective sub-themes.

#### 3.2.1. Navigating Clinical Uncertainty: From Validation to Functional Goals

Physiotherapists observed that communication within sessions enables the assessment of the specific needs of patients with FM, which in turn facilitates the collaborative establishment of therapeutic goals.

##### Clinical Validation: Addressing Patient Uncertainty and Expectations

Physiotherapists noted that, during initial sessions, many individuals with FM feel “lost”; they lack a comprehensive understanding of their condition and the underlying causes of their symptoms. This lack of clarity generates uncertainty and hinders the establishment of realistic therapeutic goals.

*‘They arrive quite lost at first. That is the reality. If I had to define them, it’s that they are lost. They don’t know exactly… many times they believe it is a degenerative disease, so that tells you everything.’*

*(PT2)*

Beyond information-giving, physiotherapists identified validation as a process of legitimacy reconstruction, enabling patients who had frequently experienced dismissal and disbelief within the healthcare system to reintegrate a coherent sense of self and illness identity.

*‘What they need, first, is to explain their situation to you and see that you understand them. There are those who need you as a guide and are already willing to make changes and start leading a more active life.’*

*(PT5)*

In fact, it is not enough for patients to understand their own condition; it is equally important that their immediate environment, such as household members or family, also understands it.

*‘Many times, they don’t feel that society, or even their most intimate environment at home (their family, friends) can truly understand them or their situation; therefore, they need to share it.’*

*(PT5)*

The discourse also revealed that many patients arrive with diverse expectations, oscillating between the hope for improvement and the demand for more traditional interventions. Some patients simply expect symptomatic relief.

*‘Patients who hope that we can help them feel better… That we give them tools.’*

*(PT1)*

Physiotherapists also described situations of limited therapeutic engagement, which may be better understood as a misalignment between patients’ expectations and the active self-management model underpinning FM rehabilitation, reflecting broader tensions within the therapeutic alliance.

*‘How do you deal with those patients? Well, I always say that a person who doesn’t want to be better won’t be better, even if you are the best place in the world. So those patients, sadly, will stay and remain in that situation.’*

*(PT2)*

These accounts also illuminate the complex socioeconomic and psychosocial factors shaping patients’ relationship with the healthcare system, including the pursuit of disability recognition as a legitimate response to years of invalidation and inadequate support.

*‘I have to say, perhaps not so much [commitment]. Most people I’ve encountered didn’t feel like working; on the contrary, they were perhaps looking for a disability status or not having to move so much.’*

*(PT6)*

One participant’s account, in particular, reflected a more skeptical stance toward disability-seeking behaviors, illustrating a tension between empathic understanding and professional skepticism that clinicians may encounter in practice (one that should be interpreted with caution rather than as representative of participants’ views as a whole).

*‘I also want to mention this because, of course, I work in an association… and the association helps them a lot with disability issues, percentages, and filing paperwork. The social worker handles much of that, and just as I have patients who truly have deep-rooted symptoms… I also have patients who take advantage of the situation and are ultimately looking for a percentage or a disability, so they don’t have to work…’*

*(PT8)*

##### Functional Meaning: Collaborative and Individualized Goal Setting

Following the initial assessment, physiotherapists establish goals tailored to the psycho-emotional profile, capabilities, and the specific stage of each person’s condition; thus, these goals must be individual in nature.

*‘Of course, they are established, but many of them… It’s quite individual.’*

*(PT1)*

Physiotherapists identified multiple dimensions that can become therapeutic goals: biomechanical factors, fatigue, sleep, pain, functionality, anxiety, depression, kinesiophobia, or catastrophizing.

*‘If you’re talking about treatment, yes, of course… but there are so many. We systematically look at the FIQ-R for pain, the VAS for fatigue, the HADS for anxiety and depression, kinesiophobia, and the SF-36 for functionality—those are the basics.’*

*(PT3)*

Functionality emerged as the most relevant goal, as it is the aspect that most impacts daily life.

*‘But basically, if I had to name a main objective, it is always functionality, functionality and, evidently, associated with a decrease in their disability. What matters is that they can increase their capacity for activities of daily living.’*

*(PT3)*

*‘By the seventh or ninth week, they tell us: “I feel better, I’m less fatigued, I can walk the dog more.” In the end, we’re talking about functionality… I’m not interested in my patient knowing how to do a bridge; I want them to manage household tasks or hold a seated posture at work for three hours without the pain they had before.’*

*(PT2)*

Physiotherapists described strategies that facilitate the setting and achievement of goals, such as breaking down large aims into small steps to make them more attainable through a graded progression.

*‘The goals were mostly set by them. I tried to be realistic… they usually seek an improvement in quality of life. If I saw that a goal was feasible, we divided it into small mini-objectives. For example, a woman wanted to walk her dogs for an hour, but she could only manage ten minutes before the pain started. So, we started with five.’*

*(PT10)*

Only a few physiotherapists mentioned using SMART criteria (Specific, Measurable, Actionable, Realistic, and Time-bound) to guide objectives toward concrete targets.

*‘To us, medium and long-term goals seem quite ambiguous or ethereal. So, we set SMART-type goals; that is, they must be related to reality and be quantifiable.’*

*(PT2)*

Long-term goals were related to an overall improvement in the person’s life, their well-being, and feeling a greater sense of control and empowerment.

*‘My long-term goal would be for them to find well-being within their body… to feel empowered within their body, which will generate empowerment in other areas of their life. My concept comes from mental health… the ultimate goal in fibromyalgia would be for the person to have power over their own life.’*

*(PT4)*

Professionals also explained that many people arrive with expectations based on traditional models (such as massages), which can hinder the establishment of functional goals.

*‘There are patients who think they are coming to receive a massage or a standard rehabilitation session. But I explain it to them. It is not only that they are informed by someone else, because they don’t quite understand it, or because perhaps the doctor once again hasn’t explained it perfectly clearly, so they believe they will receive a personalized treatment to deal with a specific point of pain.’*

*(PT5)*

Despite this, pain remained the central expectation.

*‘The first expectation patients have is that their pain will decrease. I often tell them: “Don’t focus on the pain; focus more on functionality.” Sometimes you have the same pain intensity, but you were able to walk for half an hour more… the focus is so much on the pain that other very important things are forgotten.’*

*(PT2)*

Context also influenced the process: in patient associations, there is more continuity and less pressure for immediate results, allowing for longer-term horizons.

*‘In the association, I discovered a level of tranquility… because you know it’s a patient who is used to going every week or every two weeks. That removed a lot of stress for me and allowed for longer-term treatments.’*

*(PT9)*

However, physiotherapists identified situations where active involvement was limited due to negative attitudes or apathy.

*‘Long-term goals are usually not discussed because “I have a syndrome that is a social and psychological disease”… so the attitude is often quite negative, with a lot of apathy toward doing things.’*

*(PT5)*

Fear of effort or the onset of discomfort (flare-ups) acted as a barrier, leading professionals to be extremely cautious.

*‘For those of us who don’t suffer from these problems, it’s easy to set a time-based goal… but for them, maybe not, because in two weeks a lot of things might have happened… that could even generate stress. So, it was like: “Calm down, we are on the right path,” but we move in periods of time, not on a specific day.’*

*(PT10)*

#### 3.2.2. The Therapeutic Journey: From Individual Alliance to Group Synergy

The discourses reflected that not all sessions are conceived in the same way. The first sessions with the physiotherapist tend to differ from subsequent ones. This is due to a transition in the type of therapeutic strategies and a shift from a more individualized treatment to group-based treatments.

##### The Intake Session: Building Alliance and Pain Education

Physiotherapists agreed that the first session constitutes a decisive moment. This encounter functions as a space to initiate the therapeutic relationship, gather information, manage expectations, and explain what FM is, its risks, and the rationale behind the treatment. Concepts such as the neuroscience of pain are also introduced during this stage.

*‘I dedicate the first session a bit to how can I put it locating the patient within the process of what the condition is, addressing the thought that it is a degenerative disease… /…/ What does this fibromyalgia consist of? What risks does it involve? What do you need? What are the “must-haves”. For example, I talk to them about the fact that they must make a change in their lifestyle; that if they truly want to take responsibility and have an active part in the treatment, then it is fundamental. So, the first session is basically dedicated to managing all the test results they’ve been bringing you for 10 years. All that paperwork for tests and more tests from specialists, medication… and a bit, well, providing some education and a bit of pain neuroscience as well.’*

*(PT2)*

In this first session, people with FM explain what is happening to them, how long they have been diagnosed, and how their symptoms developed. In addition to the initial anamnesis, physiotherapists perform their assessments and plan the treatment process. This planning sets the foundation for the therapeutic strategies that will be used throughout the entire treatment.

*‘In an initial session what I do is an anamnesis, and, above all, I ask: how long have they been diagnosed with fibro? How did their symptoms develop? The time from when the symptoms develop until they are diagnosed with fibro which often is quite a long period of time because they have seen many professionals like rheumatologists until they are diagnosed with fibro.’*

*(PT8)*

During the first sessions, physiotherapists explained that the objective is to convey to the individual what is happening to them and to offer strategies for application in their daily lives. These sessions typically include a dialogue component and a physiotherapy block. Throughout the first month, this work is conducted individually to prepare the person for the subsequent stages of the program.

*‘This is a part of each session. There was always a part of sophrology. A part of sophrology, then a part of discourse, which is where the perspectives from medical anthropology come in, and then a more strictly physiotherapy-related part, which I always divided into several sessions in which we did…’*

*(PT1)*

*‘So, it is true that the first few weeks, the first four sessions, constitute about a first month of preparation, approximately one month to a month and two weeks. And we treat them individually because we believe it is also important to prepare them for what is to come.’*

*(PT2)*

Individual sessions allow for a deeper understanding of each person’s particular situation and the provision of strategies to cope with pain, fatigue, or limited mobility in their daily lives. The intervention is adjusted to the patient’s current state, allowing it to be either global or focused, and more or less active depending on the individual’s needs at that moment.

*‘Currently, we do one day where I conduct individual visits, so I get to know the patients; they explain their problems to me, and I give them advice to apply in their day-to-day lives at home strategies to cope with it. The decrease in mobility, the pain, the fatigue.’*

*(PT5)*

*‘Well, it also depends on the day, but yes, usually when they come, they tell me—they explain that on that day they have more pain because we have one hour. I give them an hour and, you know, with fibromyalgia, we could start from head to toe and that’s it. But it depends. There are days when I usually spend more time on the whole back or shoulders, which also hurt them a lot; other times, the therapy is more active.’*

*(PT7)*

##### Strengthening Adherence: Transitioning Towards Peer-Supported Care

The development of adherence requires time, guidance, and progressive adjustment. Therefore, initial sessions are typically individual to build trust, resolve doubts, and reinforce the perception of self-efficacy. Once the individual begins to feel more secure and committed, they can be integrated into group programs that bolster continuity of care.

*‘So, that entire process of counseling or convincing sometimes also takes time. Therefore, the first four sessions (five including the interview) are dedicated to a more individualized treatment until the patient is more “hooked”, more adhered to what their entire context really is. And that is when we involve them within a therapeutic exercise program.’*

*(PT2)*

Physiotherapists perceived that group-based care served a function beyond logistical efficiency, operating as a space of collective validation where the shared experience of pain became normalized, reducing social isolation and transforming a profoundly private suffering into a mobilizing and identity-affirming process.

*‘So, they are out there, they have affective needs, right? And you can tell. In fact, when a group was formed, they bonded very quickly, very quickly. I mean, they started exchanging phone numbers, creating WhatsApp groups, “let’s go to lunch,” “let’s do this.”’*

*(PT4)*

*‘It’s like they are demanding love or they have that psychological need. I’m talking about how I see them, but there is a demand, and that’s why sometimes they seek out doctors, a doctor, an explanation… They demand that someone understands them. Their family want them to understand because they believe that nobody does. Their pain is like… incomprehensible. They think their pain is incomprehensible to someone who doesn’t suffer from it. That’s why, when they joined the group and began to see that they all have the same pain, or very similar pains, well, it was an explosion of euphoria; I mean, sparks flew everywhere.’*

*(PT4)*

#### 3.2.3. Therapeutic Strategies Employed

It is from the first session and throughout subsequent sessions that patients are specifically guided towards the application of various therapeutic strategies. These include PNE, cognitive behavioral therapy, mindfulness, passive strategies such as massage or radiofrequency, and active strategies including exercise, stretching, or postural patterns.

*‘It is a mix of education in a cognitive behavioral therapy session with mindfulness, some mindfulness. There are some people who use it, well, at the end, not as relaxation or as a cool-down for the therapeutic exercise part. I don’t; I do the entire 8-week program.’*

*(PT3)*

*‘What I do for them, above all, is massage and active treatment. I have Indiba radiofrequency. Are you familiar with it? No? Well, it works incredibly well for me. And afterwards, I have them do some exercises at home.’*

*(PT7)*

*‘I try to change their postural pattern a bit, because I see that the postural pattern really causes them a lot, a lot of pain, right? Well, these are pains that any of us could have if we have a very altered structural pattern with imbalances, so I try to explain it to them a bit. “Well, look, the pain in your trapezius is because you have this position. I’m going to try to open your entire anterior chain and we’re going to do posterior work.”’*

*(PT8)*

##### Understanding Pain Mechanisms as a Therapeutic Strategy

Physiotherapists identified that a fundamental therapeutic strategy is for patients to understand the mechanisms of chronic pain, enabling them to interpret their experience and recognize the actions they can undertake to improve their situation.

*‘Pedagogy in this case is important because they need to understand; they feel misunderstood, and they do not understand what is happening to them either. The problem is that it is still not fully understood. The disease does exist; it is known to exist.’*

*(PT4)*

They highlight that therapeutic education helps to give meaning to the patient’s experience and reduces the anxiety and stress associated with the diagnosis. Understanding the brain’s role in pain perception and the distinction between pain and tissue damage is considered a key component of the therapeutic process. Overall, they underscored that many patients feel misunderstood and struggle to fully comprehend what is happening to them, which reinforces the need for a clear and sustained pedagogical approach.

*‘I believe that giving them first-hand information about their process (so that they understand it and know they can do something to improve that situation) is fundamental; it significantly reduces the anxiety and stress caused by the fact that they are being diagnosed.’*

*(PT2)*

*‘There are other people who perfectly verbalize what pain neuroscience education is; they narrate it exactly, and you see that there is an absolute understanding that the brain generates the pain, that there are no “pain pathways,” that pain does not equal damage… That is why, of course, that initial understanding is important.’*

*(PT3)*

However, they also pointed out that caution is necessary, as exposure to information overload from the Internet can confuse these individuals. Therefore, it is not enough to simply provide information; it is also essential to guide them through the process of assimilating that information.

*‘I believe that information is power, and nowadays, when we are over-informed and often ill-informed, the fact that they search on their own for whatever is happening to them might induce even more panic, fear… Because they often don’t filter the information well.’*

*(PT2)*

‘*Of course, in the end, it’s fine to inform yourself, but then you have to digest all that information, and that is work you have to do. So, it wasn’t just at the exercise level; outside of that, it was a bit like: “Right, what have you done? What do you think about what we were discussing? Have you had any doubts, especially about this?”’*

*(PT10)*

##### Exercise-Based Therapeutic Strategies

Physiotherapists identified exercise as the cornerstone of FM rehabilitation, describing its progressive incorporation not merely as a physical intervention but as a process of rebuilding patients’ trust in their own bodies, gradually dismantling fear-avoidance patterns and restoring a sense of agency over physical capacity.

*‘Physical exercise is really the only thing. In fact, many of them started doing physical activity. When they finished the intervention /…/ I mean, it was incredible; the change they made in five weeks was spectacular, and what they need is physical activity, preferably in a natural environment.’*

*(PT4)*

They explained that exercise prescription is based on respecting individual tolerance, particularly in relation to pain. Physiotherapists emphasized that progression must be adapted to each person’s daily capacities, avoiding high intensities and prioritizing moderate aerobic activities. To this end, they use subjective fatigue and pain scales as practical guides. In parallel, they recommend gentle, well-tolerated exercises as a safe way to initiate the process.

*‘We progress based on tolerance; respecting the pain threshold is vital, especially in the case of people with fibromyalgia.’*

*(PT1)*

*‘And those are active strategies, above all doing cardio work, but without reaching high effort levels rather moderate, low-to-moderate at the beginning, but with the goal of reaching a moderate intensity without hitting high intensity, because otherwise, the response was not usually the best, especially in the initial phases.’*

*(PT10)*

A frequent strategy consists of starting with very low doses, even just a few repetitions, to encourage gradual and consistent exposure to exercise. Recording activities in a diary or notebook allows for an awareness of progress, reduces the sense of failure, and bolsters motivation. Observing how exercise produces less pain or fatigue over time helps to sustain long-term adherence.

*‘So, let’s lower the exercise dose even further; even if we only do three repetitions, let’s start with three, so to speak… and let’s begin to, uh, have gradual exposure to that exercise in a more or less constant way, so that little by little they can do a bit more each time.’*

*(PT10)*

*‘I tell them to keep a paper diary and record their exercises daily. I always start with very little and gradually increase the intensity or vary the exercises. Recording how many repetitions they can do before getting tired, and perceiving that it costs them less effort or they have less muscle soreness… I think it helps them feel that they are achieving it.’*

*(PT8)*

Physiotherapists underlined that adherence depends on physical activity being motivating, rewarding, and adapted to daily routines. Exercise must be something that generates interest for the individual, allowing them to maintain it without perceiving it as an obligation.

*‘I dosed the exercises for them; I assigned some that I thought could be good for their pain, but I also encouraged them not to just listen to me like, “do these exercises and you’ll be fine”, but to find something they actually like.’*

*(PT10)*

*‘The patient has to do things that fit their schedule, things they like, that fit /…/ We have to understand that adherence to all these things is practically impossible; we have to make it easy for them, so they don’t fail, so they keep doing some form of physical activity.’*

*(PT9)*

What sustains adherence, according to the physiotherapists, is the sense of well-being that exercise provides them.

*‘In the end, it’s about adherence to a physical activity that makes them feel good, where they feel motivated to keep doing it and that generates that sense of well-being.’*

*(PT4)*

It is also essential to respect rest days, preventing the individual from experiencing guilt or feeling as though they are regressing. Similarly, the aim is to promote autonomy and avoid dependency on the physiotherapist, encouraging them to manage exercise in a flexible manner.

*‘I mean, what I didn’t want was for them to depend on me to exercise. So, it’s like, “Okay, I’ll guide you. If we see each other once a week, or if not possible, once every two weeks, but you don’t have to wait for me to exercise…Because maybe the day you and I meet, you have such immense pain that you shouldn’t exercise that day. So, if we don’t do it today, in this session, it’s no big deal; do it tomorrow if you can.”’*

*(PT9)*

*‘If you feel that you have to rest that day, then rest; don’t take it the wrong way, you know? Nothing bad is going to happen, but understand that it is part of the process, you know? And don’t be scared because there are worse days.’*

*(PT10)*

Among the strategies that go beyond clinical sessions to promote pain self-regulation and the incorporation of tolerated aerobic exercise, physiotherapists recommend activities such as outdoor walking, yoga, pilates, swimming, or using a stationary bike. These are mentioned as accessible options, always prioritizing that they be comfortable, meaningful, and easy to integrate into the patient’s routine.

*‘Regarding recommendations, yes. We always tell them they have to walk for at least 40–50 min a day. I mean, the fact of leaving the house and we often tell them “leave the house, let the sun hit you, the morning sun.” I mean, that is vital and it’s extremely good.’*

*(PT2)*

*‘So now I suggest things they like, things that are, let’s say, convenient to do. For instance, if they have to commute to work, maybe I would look for some activity they can do on their way back home, so they don’t have to travel again to do an activity. Because in the end, you run out of hours, and it doesn’t get done. You can do yoga afterwards and it’s fine, Pilates, gymnastics… Unless they like any kind of class at the local council level, stretching exercises to maintain posture, back work, aqua-gym, swimming, walking, or the exercise bike.’*

*(PT5)*

##### Lifestyle-Based Therapeutic Interventions: Habits and Daily Stability

Regarding healthy habits, physiotherapists underlined that they play an essential role in FM management. Beyond nutrition, rest, and the establishment of stable routines, emphasis was placed on the importance of maintaining communication and social ties, emotional self-management, and adapting professional activity without abandoning it. These recommendations were presented as key elements to foster emotional stability, reduce the burden of pain, and improve the quality of life for those affected.

*‘Well, then I would tell you: healthy nutrition and all these approaches. Do not let yourself be defeated by the urge to isolate. Communicate, share. /…/ I always tell them that situations are what they are, and what I try to do is give them a wealth of tools from both the physiotherapy and psychology perspectives, so they can think, live, and feel differently.’*

*(PT1)*

*‘Being clear about life outside of treatment is a definite “yes”: having a very stable and calm life. That is, not having ups and downs, or people who leave you… I mean, no anger, because everything negative increases pain; leading a routine-based life.’*

*(PT6)*

*‘So, try to always go to sleep at the same time, to get up… Not doing too much every single day, which is also one of the things we might suffer from when we see an opening, it’s like “now I’m going to do everything,” and then once again, you have many days when you can’t do anything.’*

*(PT6)*

Within this framework, physiotherapists also highlighted the relevance of work activity, not so much in terms of maintaining or quitting a job, but of adapting it to each individual’s capacities and limitations. This aspect was presented as a fundamental strategy to preserve routine, a sense of purpose, and social integration, while simultaneously avoiding the isolation associated with inactivity.

*‘There are many jobs that, with minimal adaptations, allow the person to feel integrated and maintain their personal and financial contribution; for many, this is absolutely essential.’*

*(PT3)*

*‘For many of them, being able to continue working is fundamental: employment provides them with energy, dignity, and the sense of remaining active, even during periods when symptoms are most intense.’*

*(PT6)*

## 4. Discussion

This study suggests that physiotherapy in FM extends beyond the application of specific techniques or exercise programs, encompassing a more complex and evolving clinical process. Participants described an approach that begins with validating the patient’s experience, gradually shifts towards function-oriented goals, and is supported by education, exercise, and, in many cases, group-based strategies. Rather than focusing solely on symptom reduction, these findings point towards a redefinition of the physiotherapists’ role as a clinician who supports patients in making sense of their condition, promoting adherence, and fostering long-term autonomy. In this sense, the present study contributes not only new empirical knowledge but a window into the clinical enactment of the biopsychosocial model, illustrating how abstract theoretical principles are translated, adapted, and operationalized by experienced physiotherapists within the lived complexity of FM care.

A central finding of this study is the role of validation as a foundational clinical intervention in FM management. Participants consistently described how patients often arrive with uncertainty, confusion, and a lack of legitimacy regarding their symptoms, reflecting the broader characterization of FM as a “contested” condition within healthcare systems [13,37]. Previous research has highlighted that individuals with FM frequently experience invalidation and stigma, which may exacerbate distress and hinder engagement with care [27,30,38]. In this context, participants did not conceptualize validation merely as empathy but described it as an essential clinical step perceived to help reduce uncertainty and perceived threat, thereby creating the conditions they viewed as necessary for therapeutic engagement.

Before introducing exercise, education, or behavioral changes, patients need to feel understood and believed, a process that underpins the development of a strong therapeutic alliance, known to be associated with improved adherence and outcomes in chronic pain [34,39,40]. From this perspective, validation can be conceptualized as a clinical entry point that enables a transition from a fragmented illness narrative to a shared therapeutic process. This interpretation aligns with contemporary pain models emphasizing the role of perceived threat and meaning-making in symptom persistence. It also extends the previous literature by suggesting that validation may actively facilitate subsequent interventions such as PNE and graded exercise [41,42].

A second key finding relates to how physiotherapists reconceptualize therapeutic success in FM, shifting the focus from pain reduction to improvements in function, autonomy, and participation. Rather than defining progress in terms of symptom elimination, participants emphasized helping patients regain the ability to perform meaningful daily activities, tolerate effort, and re-engage with their routines, reflecting a move from a biomedical to a biopsychosocial understanding of recovery [43]. Within this framework, pain remains relevant but is no longer the sole indicator of improvement; instead, success is redefined through functional gains and a greater sense of control over the condition. This shift appears particularly important in FM, where complete pain resolution is often unrealistic, and an exclusive focus on symptom reduction may lead to frustration or perceived failure [9,18]. In this sense, physiotherapists are not positioned as professionals who “cure” the condition, but as clinicians who facilitate processes of functional adaptation and recovery of agency, supporting patients in living with greater autonomy and personal meaning, in line with self-management and empowerment approaches in chronic pain [44,45].

PNE emerged as a central component in the therapeutic process, not merely as the transmission of information but as a tool to help patients make sense of their pain, reduce fear, and decrease uncertainty. It is important to note that PNE should not be considered a stand-alone intervention or an oversimplified explanation of FM. Rather, it should be integrated within a comprehensive biopsychosocial approach that acknowledges the interaction of biological, psychological, and social factors underlying the condition. Participants emphasized that understanding pain as a process not necessarily linked to tissue damage may help break fear-avoidance cycles and facilitate engagement with movement and exercise, in line with contemporary models of chronic pain [41,42]. The previous literature has shown that reconceptualizing pain can reduce catastrophizing and kinesiophobia while improving adherence to active interventions [46,47]. Importantly, PNE did not appear as an isolated intervention, but rather as a process embedded within a validating therapeutic relationship, requiring ongoing dialogue, adaptation, and support to ensure meaningful understanding. In this sense, education seems to act as a bridge between validation and action, enabling patients to reinterpret their experience and feel more confident in re-engaging with physical activity.

Nevertheless, the findings also reveal a series of inherent tensions that merit critical consideration. First, a biomedical–biopsychosocial tension was evident in participants’ accounts. Despite adopting an explicitly biopsychosocial orientation, physiotherapists frequently encountered patients whose expectations remained anchored in passive, technique-oriented models of care (such as massage or localized treatment), reflecting the enduring dominance of biomedical frameworks in patients’ illness representations [18,34]. Second, a tension between fostering autonomy and risking dependency emerged throughout the therapeutic process: while physiotherapists consistently aimed to promote self-management and independent exercise, the relational nature of FM care and the emotional needs of this population may inadvertently reinforce reliance on the clinician as a source of validation and motivation [48]. Third, a role ambiguity tension was described, wherein physiotherapists navigated an expanded clinical identity that extended well beyond technical expertise into domains of psychological support, behavioral coaching, and social accompaniment, a role for which many reported feeling underprepared, consistent with previous findings highlighting role uncertainty in this population [31]. Fourth, physiotherapists’ occasional characterization of patients as uncommitted or motivated by secondary gain warrants critical interpretation rather than acceptance as objective fact. Such perceptions may instead reflect professional frustration after unsuccessful treatments, underrecognized depression, fatigue, or fear of symptom exacerbation, socioeconomic and occupational barriers, or a mismatch between patients’ expectations and the active self-management model promoted by clinicians. Left unexamined, these interpretations risk reproducing the very stigma that validation and legitimation practices, central to this study’s findings, are intended to counteract.

Alongside this, participants’ accounts also highlight their perception of the relevance of group-based interventions for sustaining adherence and enhancing the therapeutic process. Beyond logistical considerations, the group context was described as a space that normalizes the illness experience, reduces social isolation, and fosters identification among peers. These findings are consistent with previous research suggesting that group-based approaches in chronic pain can improve adherence, self-efficacy, and social support [30,44,49]. The transition from individual to group-based care appears to reflect a therapeutic progression, in which initial validation and individualized support create the conditions for later integration into a shared environment. Within this setting, physiotherapists described patients as not only engaging in exercise but also benefiting, in their view, from emotional support and collective motivation, suggesting that the group may be perceived to act as a therapeutic amplifier, transforming a private experience of pain and incomprehension into a shared, validated, and mobilizing process. This transition was described by a substantial number of participants, spanning public, private, and patient-association settings across different regions of Spain, indicating that this pattern is not confined to specialized or association-based programs. Nonetheless, limited availability of group programs was identified as a barrier to their wider implementation.

It is also important to situate these findings within their broader healthcare context. Participants described notably different experiences depending on their clinical setting: those working in patient associations reported greater continuity, flexibility, and reduced time pressure compared to those in public or private clinic-based practice. This variability is not trivial. The organizational conditions in which FM care takes place (including time per patient, caseload pressure, and reimbursement structures) directly shape what physiotherapists can realistically deliver. Previous research in the Spanish context has identified time constraints, high patient loads, and systemic barriers as key obstacles to implementing biopsychosocial approaches in routine practice [50,51]. These structural factors may explain, at least in part, why the comprehensive, relational, and education-based approach described by participants in this study appears more feasible in association or specialized settings than in standard public healthcare, where time per session is often severely limited.

These findings have important implications for the role of the physiotherapist in FM management, suggesting that effective care requires competencies that extend beyond the prescription of exercise or the application of physical techniques. Participants described a practice that involves clinical communication, validation, expectation management, patient education, and ongoing behavioral support, alongside the flexible dosing of exercise and a basic understanding of psychosocial factors influencing pain. It should also be acknowledged that the variability in psychological characteristics and clinical responses observed among individuals with FM is unlikely to be explained solely by motivational or psychosocial factors. Emerging evidence suggests that genetic susceptibility related to pain modulation and stress response may interact with catastrophizing, anxiety, and depressive symptoms, further supporting the need for individualized treatment within a comprehensive biopsychosocial framework [52]. This expanded role reflects the complexity of FM as a condition that cannot be adequately addressed through a purely biomedical approach [9]. In this regard, previous qualitative research has highlighted experiences of role uncertainty and perceived professional shortcomings among physiotherapists working with this population, pointing to the challenges inherent in managing such a multifaceted condition [31]. The present findings build on this perspective by illustrating how clinicians navigate this complexity in practice, not only acknowledging these challenges but also adopting concrete strategies such as validation, functional goal setting, and the integration of education and group-based approaches to support patient engagement and long-term management. In this sense, the physiotherapist’s role appears to shift from that of a technique-oriented practitioner to a clinician who facilitates understanding, supports behavioral change, and promotes autonomy within a biopsychosocial framework [39,49].

These findings have clear clinical implications for physiotherapy practice in FM. Early sessions should prioritize symptom validation as a foundational step to reduce uncertainty and facilitate engagement. Therapeutic goals may benefit from being explicitly oriented toward function, participation, and autonomy rather than pain reduction alone, aligning expectations with the fluctuating and persistent nature of the condition [9]. PNE should be delivered as an interactive and ongoing process, embedded within the therapeutic relationship, rather than as a one-time informational intervention [53]. Furthermore, a progressive transition from individual to group-based formats may enhance adherence, social support, and long-term engagement. Exercise prescription should be flexible, individualized, and meaningful, emphasizing gradual progression and self-regulation, in line with current recommendations for FM management [18]. Altogether, these findings highlight the need to strengthen training in communication skills and in the management of psychosocial complexity, while recognizing the importance of multidisciplinary collaboration for patients with more complex psychological, occupational, or social needs. Equipping physiotherapists to respond effectively to the multidimensional nature of FM addresses a challenge that has previously been associated with role uncertainty and perceived professional limitations among clinicians [31].

Taken together, the present study suggests that FM requires a model of physiotherapy that moves beyond a tissue-oriented approach toward one centered on facilitating understanding, functional recovery, and self-management. Within this framework, validation, education, and exercise do not operate as isolated components, but as interconnected elements of a coherent therapeutic process. The effectiveness of treatment appears to depend not only on what interventions are delivered, but also on how they are implemented and within which relational and contextual conditions they take place. This perspective reinforces the idea that meaningful rehabilitation in FM is grounded in the integration of evidence-based strategies with a patient-centered clinical approach.

### Limitations

This study should be interpreted considering several limitations. First, due to its qualitative design within a constructivist–interpretative framework, the findings are not intended to be statistically generalizable to all physiotherapists involved in FM care. Instead, they provide an in-depth understanding of how a specific group of experienced clinicians interprets and operates therapeutic strategies in a complex biopsychosocial condition.

Second, the use of purposive sampling combined with snowball recruitment may have favored the inclusion of participants with similar professional networks and shared clinical perspectives, particularly those aligned with a biopsychosocial approach to FM. Consequently, physiotherapists adopting more traditional biomedical models may be underrepresented, potentially influencing the prominence of themes such as validation, therapeutic alliance, and PNE. Additionally, reflexivity represents an important methodological consideration. The research team’s established biopsychosocial orientation and prior expertise in FM may have influenced both the framing of the research questions and the interpretation of findings, despite the mitigation strategies described in the reflexivity section. Additionally, social desirability bias cannot be excluded: as interviews were conducted by a researcher known to some participants through professional networks, participants may have reported practices more consistent with contemporary evidence-based ideals than with their actual clinical behavior.

Third, although the sample included professionals from different regions and healthcare settings across Spain, all participants operated within the Spanish healthcare system. Therefore, the findings are embedded within a specific sociocultural and organizational context, and their transferability to other healthcare systems should be approached with caution. Moreover, participants possessed considerable specialized experience in FM and chronic pain management (mean 11.31 years, range 1–29) and several held additional postgraduate training in this area. This level of specialization may not be representative of physiotherapists more broadly, particularly those without specific expertise in chronic pain, and should be considered when interpreting the transferability of these findings to general physiotherapy practice.

Fourth, a potential gender-related limitation should be acknowledged. While FM predominantly affects women, the sample of physiotherapists included male professionals, which may not fully capture gender-related dynamics present in clinical encounters. However, this diversity may also be considered a strength, as it allows for a broader range of professional perspectives.

Finally, the study focused exclusively on physiotherapists’ perspectives. Consequently, the therapeutic strategies and clinical interpretations presented in this study reflect professionals’ perceptions and may not fully represent the lived experiences or priorities of people with FM. Although this focus was consistent with the study’s aim of exploring an underexplored professional viewpoint, the absence of triangulation with patients’ perspectives may limit the comprehensiveness of the findings. Future qualitative studies comparing physiotherapists’ and patients’ experiences could identify areas of convergence and divergence, provide a more comprehensive understanding of FM management and further inform patient-centered physiotherapy practice.

## 5. Conclusions

This qualitative study provides in-depth insight into how physiotherapists approach the management of FM within complex and uncertain clinical contexts. The findings suggest that physiotherapy is not primarily oriented toward curing symptoms, but toward facilitating processes of understanding, adaptation, and functional recovery. Participants described a progressive clinical approach that begins with patient validation, evolves through the reorientation of therapeutic goals toward function and autonomy, and is supported by education, exercise, and, in many cases, group-based strategies.

Rather than focusing exclusively on pain reduction, physiotherapists emphasized the importance of promoting meaningful activity, self-management, and participation in daily life. Exercise was described as a central component, requiring flexible dosing and individual adaptation, while education was understood as a relational and ongoing process aimed at helping patients integrate and apply key concepts about pain. The group context emerged as a relevant therapeutic space that may enhance adherence and reduce social isolation. Overall, these findings highlight the need to understand physiotherapy in FM as a multidimensional, patient-centered process that integrates clinical knowledge with relational and contextual factors.

## Figures and Tables

**Table 1:** Semi-structured interview guide.

Section	Content/Questions
**Interview Introduction**	Thank you for agreeing to be interviewed. The purpose of this study is to explore your experience as a physiotherapist with FM patients to identify their needs and improve the care provided. Regarding consent: please remember that our conversation will be treated with strict confidentiality, and you will not be identifiable in the data reporting. Please answer the questions in your own words and feel free to share your thoughts. Do you have any questions before we begin?
**Opening Questions**	
	Tell me about the type of work you perform in your service.
	What type of patients do you treat?
	What is a typical workday like for you?
**Block 1: Patient Approach**	
	Of the patients treated at your workplace, do you know how many are diagnosed with FM?
	What does the treatment provided to FM patients consist of? What are the treatment protocols for these patients?
	Could you describe a typical treatment session?
	What do you consider a successful treatment for FM patients? What goals do you usually set in the short, medium, and long term? Why?
	Do you use active strategies during treatment? Which ones?
	What strategies do you use outside of the clinical consultation to improve your patients’ quality of life? How?
**Block 2: The Patients**	
	What do you believe helps these patients the most?
	What differences do you observe between this type of patient and others?
	In your opinion, what are FM patients trying to achieve when they seek physiotherapy?
	What are the primary needs of these patients?
	What is your background/training regarding chronic pain? How important is this training for treating these patients?
	What implications does it have for the treatment when patients understand the mechanisms of pain?
**Closing Questions**	
	Is there anything else you would like to share that we haven’t asked about?
	Thank you very much for participating in this study. I would be happy for you to contact me in the future if any further comments occur to you.

**Table 2:** Demographic and professional characteristics.

Participant ID	Gender	Age	Location	Specialized Training	Educational Level	Current Employment/Setting	Years of Practice	FM Experience
PT1	Female	52	Northeast	Physiotherapy & Anthropology	PhD	Academic Management	37	29
PT2	Female	31	Northwest	Osteopathy & Chronic Pain	PhD	University Professor & Patient Assoc. Consultant	13	9
PT3	Female	47	Northeast	Physiotherapy & Psychology	PhD	Hospital-based Physiotherapy	21	16
PT4	Female	47	Northeast	Physiotherapy & Health Sciences	PhD	Clinical Physiotherapy & University Professor	27	23
PT5	Female	37	Northeast	Occup. Therapy & Physiotherapy	BSc	Hospital (FM Unit) & Home-based Rehab	19	3
PT6	Female	36	Northeast	Mental Health Physiotherapy	MSc	Dependency Care Specialist (Hospital)	16	3
PT7	Female	45	Northeast	General Physiotherapy	BSc	Private Clinic Manager	26	26
PT8	Female	34	Northeast	General Physiotherapy	BSc	Patient Association & Private Practice	4	3
PT9	Male	45	Northwest	Kinesiology & Osteopathy	MSc	Private Practice & Patient Association	24	21
PT10	Male	36	Northeast	General Physiotherapy	MSc	Private Practice & Tele-rehabilitation	9	6
PT11	Male	30	North	General Physiotherapy	BSc	Private Practice & Tele-rehabilitation	2	1
PT12	Male	32	North	General Physiotherapy	MSc	Public Healthcare System	10	4
PT13	Male	32	North	General Physiotherapy	BSc	Public Healthcare System	10	3

**Table 3:** Themes and sub-themes organization and illustrative quotes.

Themes	Sub-Themes and Summaries	Quotes (Participant ID)
**Navigating Clinical Uncertainty: From Validation to Functional Goals**	**Clinical Validation: Addressing Patient Uncertainty and Expectations** *Summary:* Legitimize the patient’s pain to reduce uncertainty.	*‘Patients who hope that we can help them feel better… That we give them tools.’ (PT1)*
	**Functional Meaning: Collaborative and Individualized Goal Setting** *Summary:* Shifting focus from “curing” to “functioning”.	*‘The goals were mostly set by them. I tried to be realistic… they usually seek an improvement in quality of life […] a woman wanted to walk her dogs for an hour, but she could only manage ten minutes before the pain started. So, we started with five.’ (PT10)*
**The Therapeutic Journey: From Individual Alliance to Group Synergy**	**The Intake Session: Building Alliance and PNE** *Summary:* Establishing a strong therapeutic bond through listening and education.	*‘The first four sessions, constitute a first month of preparation […] we treat them individually because we believe it is also important to prepare them for what is to come.’ (PT2)*
	**Strengthening Adherence: Transitioning Towards Peer-Supported Care** *Summary:* Leveraging group dynamics to increase motivation and social belonging.	*‘[…] when a group was formed, they bonded very quickly, very quickly. I mean, they started exchanging phone numbers, creating WhatsApp groups, “let’s go to lunch,” “let’s do this.”’ (PT4)*
**Therapeutic Strategies Employed**	**Understanding Pain Mechanisms as a Therapeutic Strategy** *Summary:* De-catastrophizing pain through neuroscience education.	*‘Pedagogy in this case is important because they need to understand; they feel misunderstood […] The disease does exist; it is known to exist.’ (PT4)*
	**Exercise-based therapeutic strategies** *Summary:* Implementing graded, meaningful, and self-regulated activity.	*‘Let’s lower the exercise dose even further; even if we only do three repetitions […] little by little they can do a bit more each time.’ (PT10)*
	**Lifestyle-based therapeutic interventions: habits and daily stability** *Summary:* Stabilizing daily routines, social ties, and work adaptation.	*‘Employment provides them with energy, dignity, and the sense of remaining active, even during periods when symptoms are most intense.’ (PT6)*

## Data Availability

The data presented in this study are available upon request from the corresponding author due to privacy and ethical restrictions.

## Supplementary Materials

The following supporting information can be downloaded at: https://www.mdpi.com/article/10.3390/jcm15156099/s1, Supplementary Data S1: Consolidated criteria for reporting qualitative studies (COREQ): 32-item checklist.
