# Supplementary material for: Physiotherapists’ Clinical Reasoning and Therapeutic Strategies in Fibromyalgia: A Qualitative Interpretative Study
Source: J Clin Med. 2026 Aug 5;15(15):6099. doi: 10.3390/jcm15156099 (PMC13467318; doi:10.3390/jcm15156099)
Supplement: Supplementary file 1 [file jcm-15-06099-s001.zip › jcm-4428845-supplementary.pdf]

Supplementary Data S1: Consolidated criteria for reporting qualitative studies (COREQ): 32-item checklist

Physiotherapists' Clinical Reasoning and Therapeutic Strategies in Fibromyalgia: A Qualitative Interpretative Study

No. Item	Guide questions/description	Reported on Page #
**Domain 1: Research team and reflexivity**		
Personal Characteristics		
1. Interviewer/facilitator	Which author/s conducted the interview or focus group?	DS (Daniel Sanjuán Sánchez), lead author. Section 2.3
2. Credentials	What were the researcher’s credentials? E.g. PhD, MD	PhD candidate
3. Occupation	What was their occupation at the time of the study?	Clinical physiotherapist. Section 2.5
4. Gender	Was the researcher male or female?	Male. Not reported in the manuscript
5. Experience and training	What experience or training did the researcher have?	10 years clinical experience, 8 years in chronic pain, training in qualitative research methods. Section 2.5
Relationship with participants		
6. Relationship established	Was a relationship established prior to study commencement?	Yes, pre-existing professional relationship with some participants. Section 2.5
7. Participant knowledge of the interviewer	What did the participants know about the researcher? e.g. personal goals, reasons for doing the research	Participants were informed that the interview formed part of the interviewer's doctoral thesis research.
8. Interviewer characteristics	What characteristics were reported about the inter viewer/facilitator? e.g. Bias, assumptions, reasons and interests in the research topic	Yes, clinical standpoint, prior assumptions. Section 2.5
**Domain 2: study design**		
Theoretical framework		

9. Methodological orientation and Theory	What methodological orientation was stated to underpin the study? e.g. grounded theory, discourse analysis, ethnography, phenomenology, content analysis	Reflexive thematic analysis (Braun & Clarke), constructivist-interpretative paradigm. Section 2.1/2.4
Participant selection		
10. Sampling	How were participants selected? e.g. purposive, convenience, consecutive, snowball	Purposive + snowball (2+11). Section 2.2
11. Method of approach	How were participants approached? e.g. face-to-face, telephone, mail, email	Initial contact by email. Not reported in the manuscript
12. Sample size	How many participants were in the study?	13 physiotherapists. Section 2.2/3.1
13. Non-participation	How many people refused to participate or dropped out? Reasons?	No participants refused to participate or withdrew from the study. Not reported in the manuscript
Setting		
14. Setting of data collection	Where was the data collected? e.g. home, clinic, workplace	Face-to-face or secure video call (Teams). Section 2.3
15. Presence of non-participants	Was anyone else present besides the participants and researchers?	No; only the participant and the interviewer were present during each interview. Not reported in the manuscript
16. Description of sample	What are the important characteristics of the sample? e.g. demographic data, date	Table 2
Data collection		
17. Interview guide	Were questions, prompts, guides provided by the authors? Was it pilot tested?	No formal pilot testing was conducted. Not reported in the manuscript
18. Repeat interviews	Were repeat interviews carried out? If yes, how many?	No repeat interviews were conducted; each participant was interviewed once. Not reported in the manuscript
19. Audio/visual recording	Did the research use audio or visual recording to collect the data?	Yes, audio. Section 2.3
20. Field notes	Were field notes made during and/or after the interview or focus group?	Field notes were taken during and/or after each interview to document contextual observations. Section 2.4

21. Duration	What was the duration of the interviews or focus group?	45 to 75 min, mean 53 min. Section 2.3/3.1
22. Data saturation	Was data saturation discussed?	Yes. Section 2.2
23. Transcripts returned	Were transcripts returned to participants for comment and/or correction?	Transcripts were not returned to participants for comment or correction. Not reported in the manuscript
**Domain 3: analysis and findings**		
Data analysis		
24. Number of data coders	How many data coders coded the data?	2 (DS and EB). Section 2.4
25. Description of the coding tree	Did authors provide a description of the coding tree?	Yes. Section 2.4
26. Derivation of themes	Were themes identified in advance or derived from the data?	Derived from the data (inductive). Section 2.4
27. Software	What software, if applicable, was used to manage the data?	ATLAS.ti (web version). Section 2.4
28. Participant checking	Did participants provide feedback on the findings?	Participants did not provide feedback on the findings. Not reported in the manuscript.
Reporting		
29. Quotations presented	Were participant quotations presented to illustrate the themes/findings? Was each quotation identified? e.g. participant number	Yes, identified by ID (PT1–PT13). Results
30. Data and findings consistent	Was there consistency between the data presented and the findings?	Yes. Section 2.4
31. Clarity of major themes	Were major themes clearly presented in the findings?	Yes. Table 3. 3 themes/7 sub-themes
32. Clarity of minor themes	Is there a description of diverse cases or discussion of minor themes?	Diverse perspectives were acknowledged; for instance, one participant's account expressing skepticism toward disability-seeking behaviors was explicitly identified and discussed as an individual perception rather than representative of the sample (Section 3.2.1)
